# Trajectories of middle-aged and elderly people’s chronic diseases Disability Adjusted Life Years (DALYs): cohort, socio-economic status and gender disparities

**DOI:** 10.1186/s12939-021-01517-z

**Published:** 2021-08-03

**Authors:** Gangming Zhang, Fang Tang, Jing Liang, Peigang Wang

**Affiliations:** School of Health Sciences, Wuhan University, 115 Donghu Road, Wuhan City, Hubei Province China

**Keywords:** DALYs, Chronic diseases, Life course, SES, Gender

## Abstract

**Background:**

The accelerated aging trend brought great chronic diseases burdens. Disabled Adjusted Life Years (DALYs) is a novel way to measure the chronic diseases burden. This study aimed to explore the cohort, socioeconomic status (SES), and gender disparities of the DALYs trajectories.

**Methods:**

A total of 15,062 participants (55,740 observations) comes from China Health and Retirement Longitudinal Study (CHARLS) from 2011 to 2018. Mixed growth curve model was adopted to predict the DALYS trajectories in 45–90 years old people influenced by different birth cohorts and SES.

**Results:**

We find significant cohort, SES (resident place, education level and income) disparities differences in the chronic diseases DALYs. For individuals of earlier cohort, DALYs are developed in a late age but grow fast with age but reversed for most recent cohorts. Living in urban, having higher SES level will decrease the growth rate with age, but converges for most recent cohorts. Meanwhile, DALYs disparities of resident place and education level show gender differentials that those for female are narrowed across cohort but for male are not.

**Conclusions:**

The cohort effects on chronic diseases DALYs are accumulated with China’s unique social, and political settings. There are large inequalities in early experiences, SES and DALYs. Efforts of reducing these inequalities must focus on the lower SES individuals and those living in rural areas, which greatly benefit individuals from recent cohorts.

**Supplementary Information:**

The online version contains supplementary material available at 10.1186/s12939-021-01517-z.

## Background

Chronic disease problem of middle-aged and elderly people is an increasing serious public health challenge in China that places a heavy burden on health care [1]. According to China Center for Disease Control and Prevention (CCDC), 75.8% of the elderly population of 60 years and older in China are troubled by one or more chronic diseases, which cause a lot of physical and psychological harm to the patients due to the long course of the disease and the protracted condition, and decrease the life years [2].

Understanding the change trend of chronic diseases with age is a critical way to control them. In the former researches, morbidity and mortality are the mostly used indicators to measure the diseases [3–6]. But after Global Burden of Disease Study 2010 (GBD study 2010) published the measurement of Disabled Adjusted Life Years (DALYs) of 291 diseases and injuries, DALYs become a novel indicator to evaluate the chronic diseases. Since then, there will be measurements of chronic diseases by DALYs being published every year. However, most of them focused only one disease and analyses of the disease lack of longitudinal perspective.

As a novel health indicator, DALYs of chronic diseases may also show cohort disparities that was demonstrated having significant effects on the growth trajectories of health indicators like Self-rated Health (SRE), mental health index, Body Max Index (BMI) and index of Activities of Daily Living (ADL) [7–11]. Birth cohort is usually the represent of life course in former studies. Last century, China has witnessed a series of dramatic political, economic, and cultural upheavals, including the war (1937–1948), the great famine (1958–1961) and Cultural Revolution (1966–1976). Individuals with different birth cohort usually has different life courses, this suggests the chronic diseases DALYs of these middle-aged and elderly people may also show cohort disparities.

Besides cohort effects, previous studies also found individuals with higher socioeconomic status (SES) for example owning higher education level, living in urban area and having higher income are more likely to report better health [12–14], greater levels of physical functioning or mobility [15–17], better mental health outcomes [18–20], and lower rates of disability and mortality [21–23]. For chronic diseases, since they are closely associated with lifestyle factors like smoking, drinking and poor dietary habit [24], higher SES may have an adverse effect. When put in the context of age and life course, associations of SES and health were found gradually changed with age and interacted by cohort effect. The impact of different levels of education and income accumulates over the course of a lifetime, resulting in inequality in the health of the elderly. Zhu et al. found the advantages brought by higher education level were lager with age [25]. Chen et al. found the effect of education on health slightly decreases across successive cohorts. After the economic reform, China experienced a remarkable economic growth which improved people’s life quality and result in significant income gap. With the income gap enlarged, opportunities for people to access healthcare were also inequality. By contrast, the income gap in health trajectories diverges for earlier cohorts but converges for most recent cohorts [26]. And in the beginning of 21 century, urban residents have an advantage of 5 years in the life expectancy than rural residents as a result of inequity in acquisition of health care [27]. Li found that the urban-rural disparities of ADL and psychological wellbeing trajectories are decreased with the cohort turning younger [28]. One Japanese research found that BMI among older Japanese with higher education level was lower and it declined linearly at a faster rate over time [29]. Similarity, one Chinese research also proved that the association between education level and self-rated health has positive correlation from older cohort to younger [30]. In this way, trajectories of chronic diseases DALYs may also show SES disparities and the association between SES and DALYs may be interacted by age and cohort.

Moreover, gender disparity of health is also observed in former studies. In most countries, male life expectancy is lower than female life expectancy [31]. Crimmins et al. examined measures of ability to perform ADL and IADL functioning in 13 countries and found that the likelihood of having difficulties in carrying out daily activities and functioning problems was about 2-fold higher for women around the world [32]. For the chronic diseases, though men more likely to have heart disease, stroke, and diabetes, whereas women are more likely to have arthritis and depression [33, 34], the overall gender variations on chronic diseases are not significant [35]. however, the health disparities caused by SES and birth cohort usually show gender differences [36, 37]. This suggest DALYs of chronic diseases may also not show gender disparity but the SES disparity will be different for male and female.

Based on above, there are 3 hypothesizes in this study. Hypothesizes 1: similar to self-rated health, ADL and mental health, trajectory of DALYs of chronic diseases also have cohort disparity due to different life course. Hypothesizes 2: besides the cohort disparity, there exist SES disparities on the trajectories of chronic diseases DALYs and they will be interacted by the cohort. Hypothesizes 3: there are not gender disparity for the DALYs but the SES and cohort disparities will show gender disparity. In order to examined these hypothesizes, this study measured the DALYs for 13 chronic diseases of middle-aged and elderly people by adopting data from 4 waves of China Health and Retirement Longitudinal Study (CHARLS).

## Methods

### Data

**Fig. 1:**
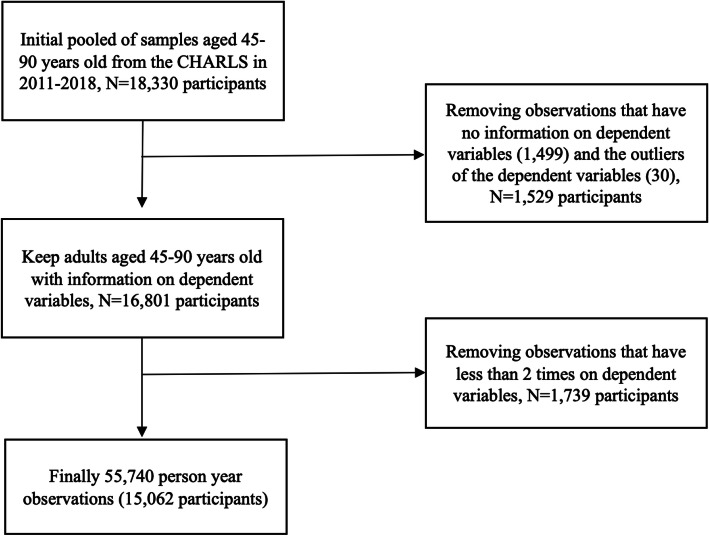
Flow diagram of the sample selection, the China Health and Retirement Longitudinal Study (2011–2018)

Data comes from China Health and Retirement Longitudinal Study (CHARLS) conducted in 2011–2018 (http://charls.pku.edu.cn/). This study adopted a four-stage, stratified, cluster sampling method to enroll province-dwelling residents from 450 villages and 150 counties in 28 provinces in China. Information was gathered using face-to-face computer-assisted personal interview (CAPI). Aims to provide comprehensive and quality data on the demographic background, family characteristics, health behavior and status, and retirement information of the middle- and old- aged residents in China. This study provides strong data support for analyzing the aging of China’s population. The age range of the sample is defined as 45–90 years old. Our sample consists of 15,101 individuals in 2011, 14,307 in 2013, 13,320 in 2015, 13,310 in 2018. Among them, individuals died by the end of 2018, and loss due to follow-up ranges from 0.07–6.8%. Thus, the sample size for analysis was 55,740 observations (15,062 participants), whose follow-up ranged between 2 and 4 waves. In this study, the DALYs measured 2 waves in 1782 participants, 3 waves in 794 participants, 4 waves in 12,525 participants. More information about the sampling framework can be seen in Fig. 1.

### DALYs

DALYs is the lost years of healthy life that caused by diseases and injures which was calculated as the sum of years of life lost (YLLs) and years lived with disability (YLDs). YLLs is the measure for the expected life years lost due to early death and YLDs is the measure for the healthy life lost living with disability and diseases. Disability weight is a critical value for the calculation of YLDs which is a measure of the level of disability of particular health state and diseases, and its values lies between 0 (nearly full health) and 1 (nearly death). In 2012, Global Burden of Disease Study 2010 (GBD 2010) published the measurement of the disability weights of 220 diseases in 195 countries or regions [38]. Then Global Burden of Disease Study 2013 (GBD 2013) further measured disability weights of 235 diseases based on the GBD 2010 [39], which is a large sample, multi-country, and multi-cultural background analysis. Thus, it has the most authoritative and universal results. In this study, valuation of disability weights for chronic diseases is based on the results of GBD 2013. In CHARLS, 14 chronic diseases were referred in the questionnaire, which are hypertension, hyperlipidemia, diabetes, cancer, chronic lung disease, hepatic disease, heart disease, stroke, kidney disease, gastric diseases, emotional and mental illness, memory related diseases, Arthritis/ rheumatism and asthma. According to Chronic Disease Death MICA-ICD-10 Codes, 13 chronic diseases are chosen in this study. Table 1 shows the valuation rules of disability weights for the 13 chronic diseases. Here are notes for the valuation rules in Table 1: (1) Wang et al. calculated the disability weight of hypertension in China based on a large-scale data from many sources [40]. (2) In 2004, WHO measured the disability weight of diabetes as 0.015 with 95%CI of 0.012 to 0.018 [41]. (3) Hepatic diseases usually include viral hepatitis, chronic hepatic diseases and hepatic cancer. In this study, we used the mean value of decompensated liver cirrhosis (the lower limit value) and viral hepatitis to measure the disability weight [42]. In this article, we regarded the YLDs as DALYs. It was because chronic diseases are always non-fatal diseases and, in our study, few participants were died during the surveys, which means the YLDs were approximately equal to the DALYs.

**Table 1:** Disability weights of 13 chronic diseases

Chronic diseases	Disability weight	Categories in GBD study/sources
Hypertension	0.36	Studies in China^a^
Diabetes	0.015	WHO studies^b^
Cancer	0.288	Cancer diagnosis and primary treatment
Chronic pulmonary disease	0.225	Moderate Chronic pulmonary disease
Hepatic disease^c^	0.123	Decompensated liver cirrhosis lower limit value
	0.006	Viral hepatitis
Heart disease	0.008	Moderate angina pectoris
Stroke	0.07	Moderate stroke
Kidney disease	0.104	Chronic kidney disease (stage 4)
Gastric diseases	0.209	Gastric bleeding lower limit value
Emotional and mental illness	0.265	Mean value of moderate anxious and depression
Memory related diseases	0.322	Mean value of moderate dementia and Parkinson’s disease
Arthritis/ rheumatism	0.080	Mean value of moderate musculoskeletal disorders
Asthma	0.036	Partly controlled asthma

Notes:

^a^ Wang et al. calculated the disability weight of hypertension in China based on a large-scale data from many sources

^b^ In 2004, WHO measured the disability weight of diabetes as 0.015 with 95%CI of 0.012 to 0.018

^c^ Hepatic diseases usually include viral hepatitis, chronic hepatic diseases and hepatic cancer. In this study, we used the mean value of decompensated liver cirrhosis (the lower limit value) and viral hepatitis to measure the disability weight

### Independent variable

#### Birth cohort

Birth cohort represented the social set to some degree. In this study, birth cohort was used to reflect the variant life course. In order to distinguish the life course, this study established the 1942–1946, 1947–1953, 1954–1959 and 1960–1964 these four birth cohorts which had distinctive life course, detail division basis on supplementary material Table S1. For a convenience of analysis, birth cohort was regarded as continuous variable which were valued as 1 to 4 for 1942–1947, 1948–1953, 1954–1959 and 1960–1964.

#### Socioeconomic status

In this study, we use three different measures of SES: educational level, resident place, and per capita family income. To simplify the interpretation, we use three dichotomous variables for education level in the baseline survey. We operationalize educational level as those with illiterate (=1), those with elementary school (=2), those with more than middle school (=3). Since the research subjects are all middle-aged and elderly, the educational level will not change significantly, the educational level of this study mainly used the educational level of the respondents in the baseline survey. We operationalize urban and rural residency as those who living in urban (=0), those who living in rural (=1). The income variable is the per capita income of the respondent’s household, and the logarithm was used to avoid the influence of extreme values.

#### Control variable

In order to analyze the cohort effect and the influence of socioeconomic status more accurately, other characteristics may affect the middle-aged and elderly health should to be controlled. The CHARLS provides information on individual characteristics variables, including gender, and marital status. Since chronic diseases are also significantly related to the status of receiving medical services, to control for potential medical service variation and health behaviors, we controlled the effects of the inpatient services expenditures and outpatient services expenditures, health behaviors (drinking and smoking), and died/loss to follow-up with dummy variables, using interquartile range to determine the second quartile, expenditures<the second quartile (=0), expenditures > = the second quartile (=1), drinking (=1), not drinking(=0), smoking(=1), not smoking (=0), died/loss to follow-up(=1). At the same time, the self-expense ratio in outpatient and inpatient services were also included as a control variable in this study. Table 1 presents baseline characteristics of the population-based sample and analysis sample.

### Statistical analysis

YLDs of 13 chronic diseases are calculated firstly.

1 $$ {YLDs}_j={W}_j\ast {T}_j $$

$$ {YLDs}_{all}=\sum \limits_{j=1}^{13}{YLDs}_j(2) $$

In Eq. (1), *W*_*j*_ is the disability weight of *j*_*th*_ chronic disease. *T*_*j*_ is the years having the disease which can be calculated.

Then the Hierarchical growth curve model is used to analyze the longitudinal data. This modeling reveals significant cohort variations in the age trajectories of health [26]. Most researches illustrated that hierarchical linear model (HLM) or growth curve models can be used to test for cohort differences in age trajectories [43, 44]. And our study figured out the fitting indicators of models (the BIC, AIC, −2LL), found that the quadratic curve fits DALYs change better than simple linear or other curves (See supplementary material Table S2). The hierarchical growth curve model constructed in this study has two levels. The level-1 is repeated measurements within the individual. In this article, it corresponds to the DALYs tracking measurement values of a middle-aged and elderly at different ages. These measurements are nested in the data structure of different middle-aged and elderly in the level-2:

Level-1:

3 $$ {y}_{ij}={\beta}_{0j}+{\beta}_{1j}\ast {age}_{ij}+{\beta}_{2j}\ast age\hat{\mkern6mu} {2}_{ij}+{e}_{ij} $$

In this model, *j* represents the individuals from 1, ⋯, *N* samples. *y*_*ij*_ represents the DALYs of individual *j* at age *i*. We center the age variable around, the median age of the cohort to which person *i* belongs, which can eliminate confounding of age and cohort variables. Age represents the median age *i* of individual *j*. Age^2 is the median age square *i* of individual *j*. A quadratic term could explain the potential nonlinear effects of age growth. *β*_0*j*_ represents the initial value of DALYs at the median age. Other control variables of are included in the outcome parameter model; *β*_1*j*_ and *β*_2*j*_ represent the slopes of individual DALYs with the measured median age, and *e*_*ij*_ represents the residual error of an individual *j* at median age *i*.

Level-2:

Intercept parameter

4 $$ {\beta}_{0j}={r}_{00}+{r}_{01}\ast {cohort}_j+{u}_{0j} $$

Slope parameter

5 $$ {\beta}_{1j}={r}_{10}+{r}_{11}\ast {cohort}_j+{r}_{12}\ast {SES}_j+{r}_{13}\ast {cohort}_j\ast {SES}_j+{u}_{1j} $$

6 $$ {\beta}_{2j}={r}_{20}+{r}_{21}\ast {cohort}_j+{r}_{22}\ast {SES}_j+{r}_{23}\ast {cohort}_j\ast {SES}_j+{u}_{2j} $$

The aim of the level-2 analysis is to research heterogeneity in change across individuals and to determine the association between predictors (SES and cohort) and the shape of each person’s growth trajectory. *β*_0*j*_ called fixed-effect model parameter, represents the influence of cohort characteristics on the intercept in this modeling. The *r*_01_ represents the interaction effect between cohort and median age. *r*_10_ , *r*_11_, *r*_12_ and *r*_13_ are coefficients for the parameters of cohort, socioeconomic status, socioeconomic status × cohort on the slope of DALYs, means the interaction effects of these variables with median age. *r*_20_ , *r*_21_, *r*_22_ and *r*_23_ represent the parameters of cohort, socioeconomic status, socioeconomic status × cohort on the slope of DALYs, means the interaction effects of these variables with the square of median age. *u*_0*j*_ 、 *u*_1*j*_ and *u*_2*j*_ are random effects of intercept and slope parameters. Other control variables of individual characteristics that do not change with age or time, such as gender, education level, etc., are placed in the level-2 model. Because people who stay in the sample may be healthier, vulnerable groups are more likely to die early, and the impact of socioeconomic status on health may be overestimated. A simple but effective solution, which is to classify the loss types directly in the level-2 model. The specific approach is entering the dummy variables of death and loss to follow-up (=1) [26], we account for the possibility that those who lost responders due to death or non-response will have high DALYS than survivors with complete data. In addition, for the absence of independent variables, we use multiple imputation methods. All the model were estimated using SAS 9.4. The statistical significance was set at α = 0.05.

## Results

Table 2 presents the descriptive statistics of dependent and independent variables tabulated by cohorts. The participants for male and female were 7146 (47.31%) and 7949 (52.66%), respectively. Chronic diseases DALYs for the four cohorts all showed an increasing trend from 2011 to 2018. Participants living in the rural (80.01%) are more than those living in urban (19.99%). With cohorts turning younger, illiterate rate are decreasing and the house income is rising.

**Table 2:** Descriptive Statistics of All Variables in the Analyses

Variables	1942–1947	1948–1953	1954–1959	1960–1964	All
**Disease Burden [Mean (SD)]**					
DALYs (2011)	1.46 (2.74)	1.25 (2.48)	0.95 (2.03)	0.73 (1.60)	1.10 (2.37)
DALYs (2013)	2.54 (3.65)	2.14 (3.25)	1.63 (2.61)	1.28 (2.16)	1.89 (3.09)
DALYs (2015)	3.00 (3.99)	2.54 (3.56)	1.94 (2.90)	1.57 (2.50)	2.23 (3.36)
DALYs (2018)	3.24 (4.18)	2.75 (3.76)	2.10 (3.07)	1.72 (2.67)	2.41 (3.55)
**Gender [N (%)]**					
female	806 (46.69)	1623 (50.62)	1511 (50.37)	1545 (53.98)	7949 (52.66)
male	816 (50.31)	1583 (49.38)	1498 (49.63)	1317 (46.02)	7146 (47.31)
**SES-Resident place [N (%)]**					
Urban	363 (22.38)	650 (20.27)	552 (18.39)	521 (18.20)	3019 (19.99)
Rural	1259 (77.62)	2557 (79.73)	2449 (81.61)	2342 (81.80)	12,082 (80.01)
**SES-education [N (%)]**					
Illiterate	490 (30.27)	943 (29.41)	747 (24.92)	358 (12.5)	4071 (26.99)
Elementary school	753 (46.51)	1598 (49.84)	1111 (37.07)	904 (31.58)	6045 (40.07)
Middle school and higher	376 (23.22)	665 (20.74)	1139 (38.00)	1601 (55.92)	4970 (32.94)
**SES-Family income [Mean (SD)]**					
income (log)	4.17 (0.61)	4.29 (0.59)	4.41 (0.59)	4.54 (0.52)	4.33 (0.62)
**Marriage status [N (%)]**					
have spouse	1298 (80.02)	2683 (83.66)	2550 (84.97)	2447 (85.47)	12,333 (81.67)
no spouse	324 (19.98)	524 (83.66)	451 (15.03)	416 (14.53)	2768 (18.33)
**Medical variables [Mean (SD)]**					
Hospitalization OOP percent	0.21 (0.40)	0.38 (10.77)	0.16 (0.34)	1.335 (6.47)	1.114 (6.53)
Doctor visit OOP percent	0.37 (0.48)	0.63 (12.72)	0.35 (0.49)	0.37 (0.63)	0.42 (6.80)
**Hospitalization OOP [N (%)]**					
Q1	1087 (67.02)	2268 (70.72)	2277 (75.87)	2312 (80.75)	11,097 (73.49)
Q2	535 (32.98)	939 (29.28)	724 (24.13)	551 (19.25)	4004 (26.51)
**Hospitalization total fee [N (%)]**					
Q1	1065 (65.66)	2243 (69.94)	2255 (75.14)	2289 (79.95)	10,942 (72.46)
Q2	557 (34.34)	964 (30.06)	746 (24.86)	574 (20.05)	4159 (27.54)
**Doctor visit OOP [N (%)]**					
Q1	894 (55.12)	1819 (56.72)	1799 (59.95)	1703 (59.48)	8794 (58.23)
Q2	728 (44.88)	1388 (43.28)	1202 (40.05)	1160 (40.52)	6307 (41.77)
**Doctor visit total fee [N (%)]**					
Q1	871 (53.7)	1792 (55.88)	1777 (59.21)	1683 (58.78)	8669 (57.41)
Q2	751 (46.3)	1415 (44.12)	1224 (40.79)	1180 (41.22)	6432 (42.59)
**Died**					
died = 1	148 (7.14)	124 (3.69)	70 (2.06)	39 (1.12)	729 (4.83)
not died = 0	1924 (92.86)	3235 (96.31)	3327 (97.94)	3438 (98.88)	14,372 (95.17)
**Smoking**					
smoking = 1	879 (42.42)	1378 (41.04)	1402 (41.32)	1230 (35.42)	4889 (39.76)
Not smoking = 0	1193 (57.58)	1980 (58.96)	1991 (58.68)	2243 (64.58)	7407 (60.24)
**Drinking**					
drinking = 1	654 (30.31)	748 (24.36)	749 (24.45)	720 (22.82)	2871 (25.08)
drinking = 0	1504 (69.69)	2323 (75.64)	2315 (75.54)	2435 (77.78)	8577 (74.92)

**Fig. 2:**
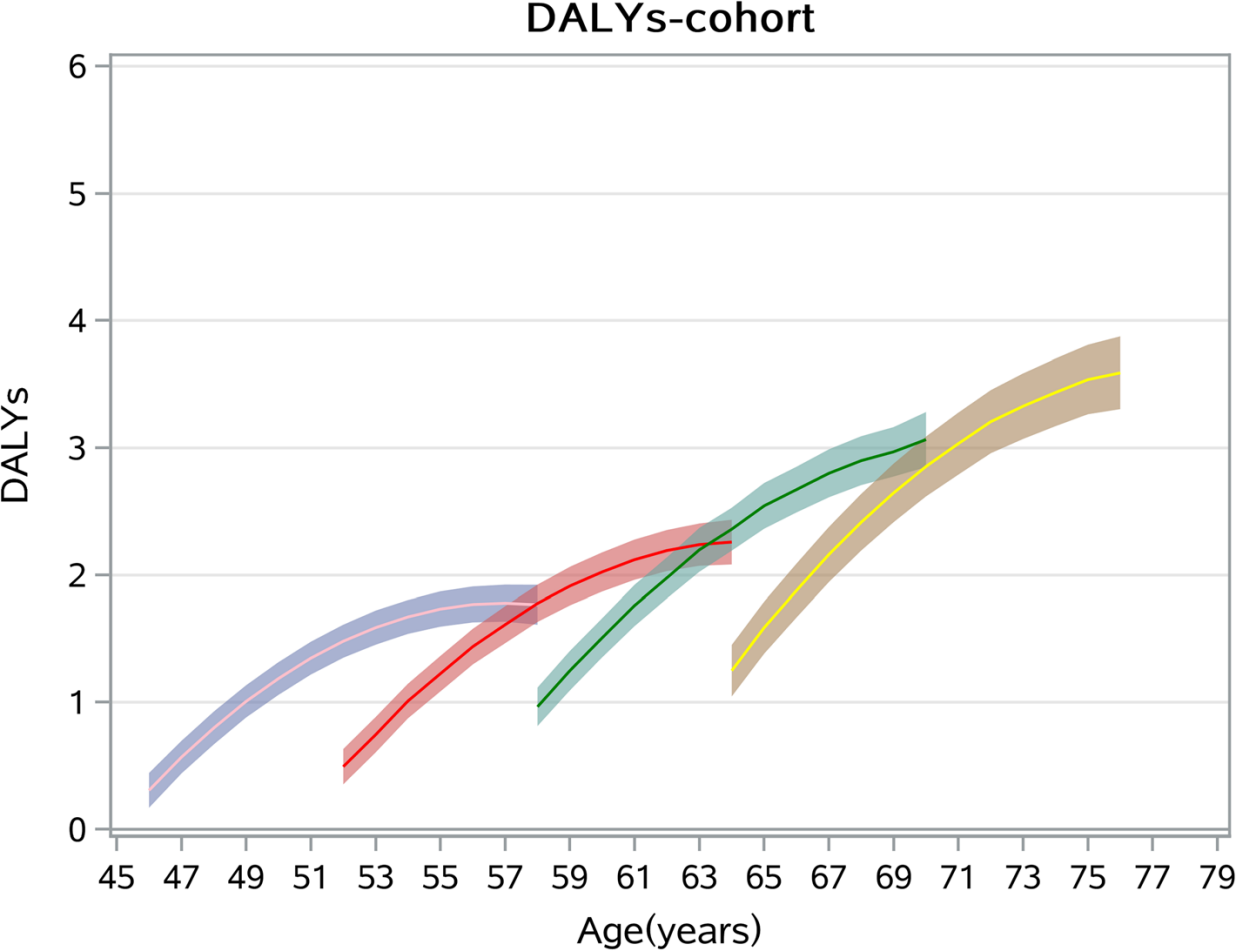
Cohort disparity of chronic diseases DALYs trajectories

Table 3 presents findings from the estimation of growth curve models. We conducted analysis of cohort, SES indicators (resident place, income and education level) and the interactions of SES-by-cohort from Model 1 to Model 3. Model 1 strongly support Hypothesis 1: there exist significant cohort differentials in trajectories of middle-aged and elderly people’s chronic diseases DALYs. Model 1 shows the age trajectory of DALYs after controlling the cohort effect, which has a mean of 1.55 (95%CI: 1.086, 2.014) years and increases as a rate of 0.49 (95%CI: 0.469, 0.511) years per year of age, slowing at a rate of 0.005 with age, thereby exhibiting a quadratic pattern. Results also shows there are strongly significant cohort variations in the intercept or mean level of DALYs. Respondents in younger cohorts has a 0.232 (95%CI: − 0.305, − 0.16) years less than older cohorts for the DALYs intercept and its growth with age show less rapid ((the cohort-by-age interaction is − 0.143 with 95% CI: − 0.152, − 0.134). For a better view of these trajectories, based on estimates of Model 1 we plotted Fig. 2 in below. From Fig. 2, it can be found that older cohorts have a faster growth of DALYs with age, however, chronic diseases of older cohorts seem appear in a later age than younger cohorts.

**Table 3:** Cohort and socioeconomic disparities of chronic diseases DALYs

	Model 1		Model 2		Model 3	
Variables	***β***(95%CI)	***P***	***β***(95%CI)	***P***	***β***(95%CI)	***P***
**Fixed effect**						
Intercept	1.55 (1.086, 2.014)	<.0001	2.449 (1.795, 3.102)	<.0001	1.882 (1.192, 2.571)	<.0001
Age	0.49 (0.469, 0.511)	<.0001	0.575 (0.529, 0.621)	<.0001	0.982 (0.821, 1.142)	<.0001
Age^2	−0.005(− 0.006, − 0.005)	<.0001	− 0.004(− 0.006, − 0.001)	0.002	−0.013(− 0.02, − 0.006)	<.0001
Age*Cohort	− 0.143(− 0.152, − 0.134)	<.0001	− 0.138(− 0.147, − 0.129)	<.0001	− 0.315(− 0.381, − 0.248)	<.0001
Age*Edu Illiterate	− 0.002(− 0.002, − 0.001)	<.0001	0.035 (0.021, 0.049)	<.0001	0.128 (0.078, 0.178)	<.0001
Age*Edu Elementary school			0.028 (0.015, 0.04)	<.0001	0.11 (0.065, 0.154)	<.0001
Age*Middle school and higher (Reference)			0(.,.)	.	0(.,.)	.
Age*Hukou Urban			−0.037(− 0.051, − 0.024)	<.0001	− 0.135(− 0.186, − 0.084)	<.0001
Age*Lg-income			− 0.021(− 0.029, − 0.012)	<.0001	−0.108(− 0.141, − 0.076)	<.0001
Age*Cohort*Edu Illiterate					−0.041(− 0.062, − 0.021)	<.0001
Age*Cohort*Edu Elementary school					−0.034(− 0.052, − 0.016)	<.0001
Age*Cohort*Middle school and higher (Reference)					0(.,.)	.
Age*Cohort*Hukou Urban					0.039 (0.018, 0.059)	<.0001
Age*Cohort*Lg-income					0.039 (0.025, 0.052)	<.0001
Age^2*Cohort	−0.001(− 0.002, − 0.001)	<.0001	− 0.002(− 0.002, − 0.001)	<.0001	−0.003(− 0.006, − 0.001)	0.006
Age^2*Edu Illiterate			0.001(−0.00006, 0.001)	0.07	−0.001(− 0.003, 0.002)	0.522
Age^2*Edu Elementary school			0.0002(− 0.0004,0.0009)	0.478	− 0.002(− 0.004, 0)	0.052
Age^2*Edu Middle school and higher (Reference)			0(.,.)	.	0(.,.)	.
Age^2*Hukou Urban			0.000236(− 0.00048, 0.001)	0.521	0.004 (0.002, 0.006)	0.001
Age^2*Lg-income			−0.001(− 0.001, − 0.00016)	0.008	0.001 (0, 0.003)	0.117
Age^2*Cohort*Edu Illiterate					−0.001(− 0.001, 0)	0.082
Age^2*Cohort*Edu Elementary school					−0.00008(− 0.001, 0.001)	0.808
Age^2*Cohort*Middle school and higher (Reference)					0(.,.)	.
Age^2*Cohort*Hukou Urban					− 0.0003(− 0.001, 0.000453)	0.436
Age^2*Cohort*Lg-income					0.001 (0.000039, 0.001)	0.034
Died	−0.108(−0.469, 0.254)	0.56	−0.1(− 0.462, 0.261)	0.586	− 0.083(− 0.444, 0.279)	0.654
Female	− 0.011(− 0.148, 0.126)	0.874	−0.007(− 0.143, 0.129)	0.92	0.000423(− 0.136, 0.137)	0.995
Hukou Urban	−0.228(− 0.358, − 0.098)	0.001	− 0.666(− 0.867, − 0.464)	<.0001	−0.541(− 0.753, − 0.328)	<.0001
Edu Illiterate	− 0.088(− 0.224, 0.049)	0.207	0.299 (0.092, 0.505)	0.005	0.155(− 0.063, 0.372)	0.164
Edu Elementary school	0.031(−0.083, 0.145)	0.593	0.342 (0.161, 0.522)	0	0.216 (0.023, 0.41)	0.028
Edu Middle school and higher (Reference)	0(.,.)	.	0(.,.)	.	0(.,.)	.
Lg-income	−0.014(− 0.097, 0.069)	0.735	− 0.235(− 0.363, − 0.107)	0	−0.117(− 0.251, 0.018)	0.09
Cohort	−0.232(− 0.305, − 0.16)	<.0001	−0.301(− 0.375, − 0.226)	<.0001	−0.321(− 0.396, − 0.247)	<.0001
Hospitalization OOP percent	0.00002(− 0.0003, 0.0004)	0.896	0.00002(− 0.0003, 0.00036)	0.89	0.00003(− 0.00032, 0.00036)	0.887
Doctor visit OOP percent	0.007 (0.002, 0.013)	0.01	0.007 (0.002, 0.013)	0.011	0.007 (0.002, 0.013)	0.01
Hospitalization OOP Q2	−0.116(−0.503, 0.271)	0.557	−0.118(− 0.505, 0.268)	0.548	−0.14(− 0.526, 0.247)	0.479
Hospitalization total fee Q2	−0.038(− 0.422, 0.345)	0.845	−0.033(− 0.416, 0.35)	0.867	−0.01(− 0.393, 0.374)	0.961
Doctor visit OOP Q2	−0.276(− 0.463, − 0.09)	0.004	−0.276(− 0.462, − 0.09)	0.004	−0.271(− 0.457, − 0.085)	0.004
Doctor visit total fee Q2	0.042(− 0.141, 0.225)	0.649	0.042(− 0.141, 0.225)	0.653	0.032(− 0.151, 0.215)	0.73
No spouse	0.13 (0.002, 0.259)	0.047	0.128 (0, 0.257)	0.051	0.127(−0.002, 0.255)	0.054
Drinking	0.002(−0.125, 0.129)	0.973	0.001(−0.126, 0.128)	0.991	0.002(−0.125, 0.129)	0.977
Smoking	−0.045(− 0.183, 0.092)	0.518	− 0.046(− 0.183, 0.092)	0.517	−0.05(− 0.187, 0.088)	0.481
**Random effect**						
Intercept variance	10.34 (0.172)	<.0001	10.314 (0.171)	<.0001	10.291 (0.171)	<.0001
Slope variance	0.035 (0.001)	<.0001	0.035 (0.001)	<.0001	0.035 (0.001)	<.0001
Co-variance	0.498 (0.01)	<.0001	0.496 (0.01)	<.0001	0.496 (0.01)	<.0001
Residual	0.209 (0.003)	<.0001	0.209 (0.003)	<.0001	0.207 (0.003)	<.0001
-2LL	89,396.3		89,377.3		89,361.5	
AIC	89,404.3		89,385.3		89,369.5	
BIC	89,432.5		89,413.5		89,397.7

**Fig. 3:**
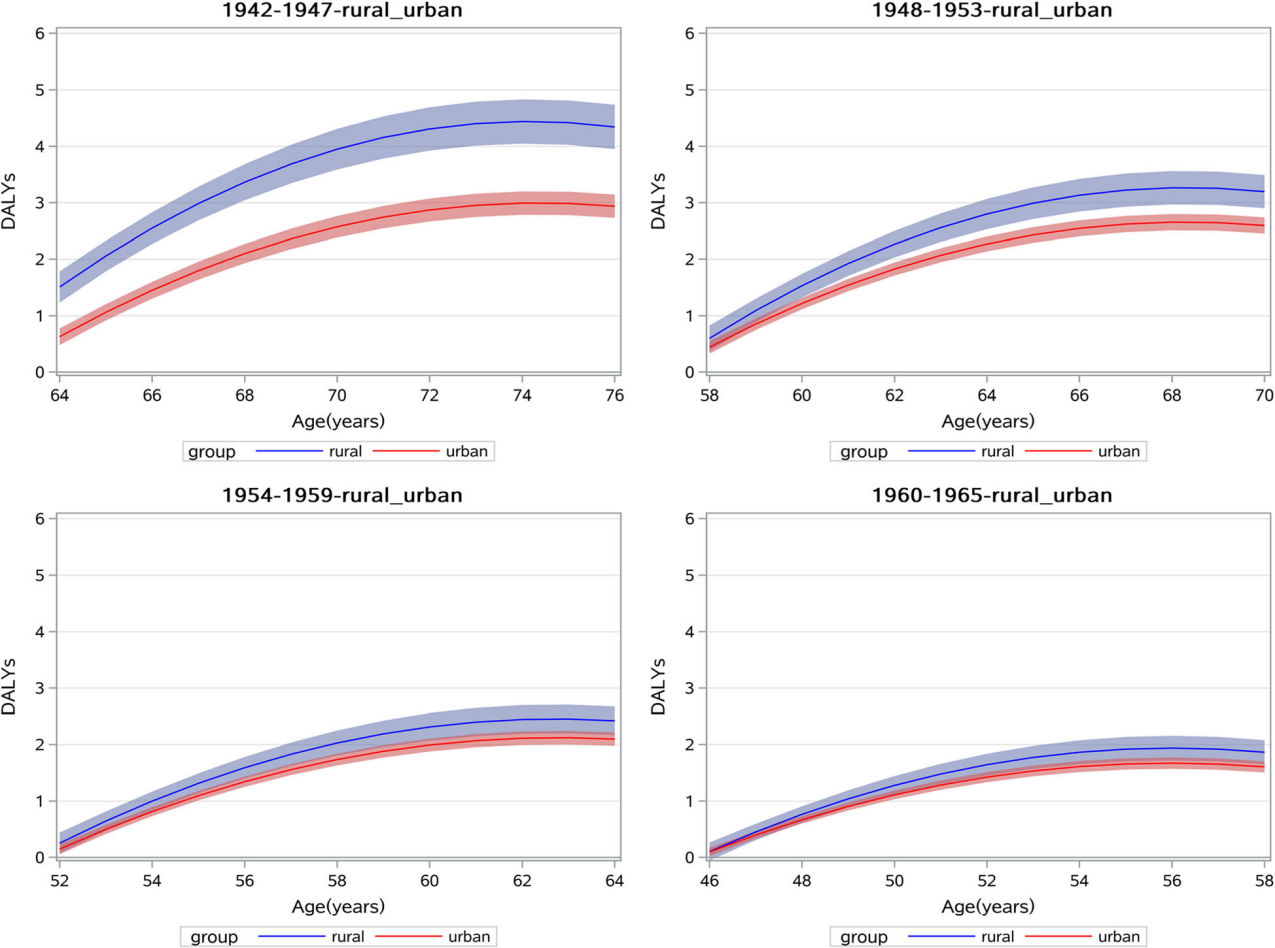
Resident place disparity of chronic diseases DALYs

Model 2 and 3 show findings support for Hypothesis 2: SES would affect the growth trajectories of DALYs and it was interacted by cohort effect. Model 2 tests the SES indicators disparities of DALYs age trajectories and find there exist significant SES gradients in the intercept or mean level of DALYs. Relative to the most disadvantaged (individuals whose education level were illiterate and elementary school), respondents with middle school and higher education levels have less DALYs by 0.299 (95%CI:0.092,0.505) and 0.342 (95%CI:0.161,0.522) years and its growth trend with age was slower (the age-by-education level interaction is 0.035 with 95%CI: 0.021, 0.049 and 0.028 with 95%CI: 0.015, 0.04). Compared with individuals living in rural area, respondents living in urban have a lower DALYs by 0.666 (95%CI: − 0.867, − 0.464) years and its growth with age is also slower (the age-by-resident place interaction is − 0.037 with 95%CI: − 0.051, − 0.024). Consistent with resident place and education level, respondents with higher income have lower DALYs by 0.235 (95%CI: − 0.363, − 0.107) years by one unit, and its age growth trajectory still show less rapid (the age-by-income interaction is − 0.021 with 95%CI: − 0.029, − 0.012). Model 3 added interactions of SES indicators by cohort to explore the SES differentials across cohorts. Model 3 reports the significant age-cohort-SES indicators interactions which suggest the DALYs SES disparities have differentials across cohorts. The age-by-resident place-by-cohort interaction is 0.039 (95%CI: 0.018,0.059). This indicate the DALYs disparities of resident place are narrowed with cohort turning younger. Similarly, we find the education level and income disparities of DALYs are also narrowed with cohort turning younger (the age-by-education level-by-cohort interaction is − 0.041 and − 0.034 with 95%CI: − 0.062, − 0.021 and − 0.052, − 0.016; the age-by-income-by-cohort interaction is 0.039 with 95%CI: 0.025,0.052). For a better interpret of these trajectories, we plotted age trajectories of DALYs by resident place in Fig. 3. From Fig. 3, we can find DALYs growth trajectory of urban respondents are slower than those live in rural and resident place differentials are narrowed in younger cohorts.

Table 4, which mainly support Hypothesis 3, presents the cohort and SES disparities of DALYs across gender by conducting analyses for male and female separately. From the results, it is found to be differences in growth trajectories of DALYs for respondents with different cohort and SES indicators across gender. Specifically, disparities of DALYs growth trajectories for male caused by education level and resident place will not be narrowed with cohort turning younger (the age-by-education level-by-cohort interaction and age-by-resident place interaction are not significant). On the contrary, these disparities are narrowed for female with cohort turning younger (the cohort-by-age-by-education level interaction are − 0.054 with 95%CI: − 0.102,0.006 and − 0.042 with 95%CI: − 0.007, − 0.015 and the age-by-resident place-by-cohort interaction are 0.075 with 95%CI: 0.041,0.11).

**Table 4:** Cohort and SES disparity of chronic diseases DALYs across gender

	Male		Female	
Variables	***β***(95%CI)	***p***	***β***(95%CI)	***p***
**Fixed effect**				
Intercept	1.048 (0.148, 1.948)	0.023	2.137 (0.896, 3.377)	0.001
Age	0.978 (0.781, 1.175)	<.0001	1.03 (0.748, 1.311)	<.0001
Age^2	−0.015(−0.023, −0.006)	0.001	−0.01(−0.023, 0.002)	0.115
Age*Cohort	−0.317(−0.399, − 0.236)	<.0001	−0.325(− 0.441, − 0.21)	<.0001
Age*Edu Illiterate	0.09 (0.026, 0.155)	0.006	0.153 (0.038, 0.267)	0.009
Age*Edu Elementary school	0.086 (0.025, 0.147)	0.006	0.127 (0.06, 0.195)	0.0002
Age*Middle school and higher (Reference)	0(.,.)	.	0(.,.)	.
Age*Hukou Urban	−0.064(−0.13, 0.001)	0.055	−0.24(− 0.322, − 0.158)	<.0001
Age*Lg-income	− 0.116(− 0.156, − 0.077)	<.0001	−0.1(− 0.157, − 0.044)	0.001
Age*Cohort*Edu Illiterate	−0.026(− 0.052, − 0.00025)	0.048	−0.054(− 0.102, − 0.006)	0.027
Age*Cohort*Edu Elementary school	−0.024(− 0.049, 0.000488)	0.055	−0.042(− 0.07, − 0.015)	0.003
Age*Cohort*Middle school and higher (Reference)	0(.,.)	.	0(.,.)	.
Age*Cohort*Hukou Urban	0.014(−0.013, 0.041)	0.321	0.075 (0.041, 0.11)	<.0001
Age*Cohort*Lg-income	0.042 (0.026, 0.059)	<.0001	0.035 (0.011, 0.058)	0.003
Age^2*Cohort	−0.003(−0.006, − 0.00005)	0.046	− 0.004(− 0.008, − 0.0002)	0.04
Age^2*Edu Illiterate	0.001(− 0.003, 0.004)	0.7	−0.003(− 0.008, 0.001)	0.175
Age^2*Edu Elementary school	−0.001(− 0.004, 0.002)	0.608	−0.003(− 0.007, − 0.00029)	0.032
Age^2*Middle school and higher (Reference)	0(.,.)	.	0(.,.)	.
Age^2*Hukou Urban	0.002(−0.002, 0.005)	0.323	0.007 (0.004, 0.011)	<.0001
Age^2*Lg-income	0.002 (0.000159, 0.004)	0.032	−0.00023(− 0.003, 0.002)	0.859
Age^2*Cohort*Edu Illiterate	−0.001(− 0.002, 0.000237)	0.138	0.000031(− 0.002, 0.002)	0.972
Age^2*Cohort*Edu Elementary school	−0.00021(− 0.001, 0.001)	0.658	0.000026(− 0.001, 0.001)	0.96
Age^2*Cohort*Middle school and higher (Reference)	0(.,.)	.	0(.,.)	.
Age^2*Cohort*Hukou Urban	−0.00008(− 0.001, 0.001)	0.87	− 0.001(− 0.002, 0.001)	0.325
Age^2*Cohort*Lg-income	0.000364(− 0.00021, 0.001)	0.211	0.001(− 0.000003, 0.002)	0.051
Died	−0.093(− 0.633, 0.448)	0.737	− 0.051(− 0.537, 0.435)	0.836
Hukou Urban	− 0.496(− 0.775, − 0.217)	0.001	−0.632(− 0.965, − 0.298)	0.0002
Edu Illiterate	0.183(− 0.099, 0.464)	0.204	−0.088(− 0.527, 0.351)	0.694
Edu Elementary school	0.204(−0.067, 0.475)	0.141	0.216(−0.062, 0.495)	0.128
Edu Middle school and higher (Reference)	0(.,.)	.	0(.,.)	.
Lg-income	−0.113(−0.281, 0.056)	0.19	−0.133(− 0.358, 0.092)	0.248
Cohort	−0.343(− 0.438, − 0.249)	<.0001	−0.278(− 0.402, − 0.155)	<.0001
Hospitalization OOP percent	−0.00009(− 0.0004, 0.0003)	0.617	0.001 (0.000033, 0.002)	0.044
Doctor visit OOP percent	0.007 (0.002, 0.013)	0.011	0.114(−0.535, 0.763)	0.731
Hospitalization OOP Q2	0.288(−0.177, 0.752)	0.225	−0.178(− 0.876, 0.519)	0.617
Hospitalization total fee Q2	−0.133(− 0.593, 0.328)	0.573	0.308(− 0.384, 1.0003)	0.382
Doctor visit OOP Q2	0.129(−0.095, 0.353)	0.259	0.452(−0.096, 1.001)	0.106
Doctor visit total fee Q2	0.099(−0.122, 0.321)	0.38	−0.293(− 0.67, 0.084)	0.128
No spouse	0.207 (0.051, 0.363)	0.009	−0.042(− 0.272, 0.188)	0.719
Drinking	0.01(−0.193, 0.212)	0.924	0.04(−0.129, 0.208)	0.643
Smoking	0.261 (0.029, 0.493)	0.028	−0.209(− 0.386, − 0.032)	0.021
**Random effect**				
Intercept variance	10.634 (0.22)	<.0001	9.675 (0.27)	<.0001
Slope variance	0.036 (0.001)	<.0001	0.034 (0.001)	<.0001
Co-variance	0.521 (0.013)	<.0001	0.448 (0.015)	<.0001
Residual	0.214 (0.003)	<.0001	0.192 (0.004)	<.0001
-2LL	58,933.6		30,622.6	
AIC	58,941.6		30,630.6	
BIC	58,968.1		30,654.5	

## Discussion

The objective of this study is to explore the growth trajectory of DALYs of chronic diseases for middle-aged and elderly people and its disparities across cohort, SES (resident place, income and education level) and gender. We find there are significant cohort and SES disparities in the growth trajectories and the SES and cohort disparities will show differentials across different gender.

In this study, we calculate the chronic diseases DALYs by valuing the disability weights from GBD study 2013. This is a creative application for the GBD study which is firstly used in the exploration of early psychological risks effects on health [45], and the results of this study further proved the validity of this methods.

Our findings suggest that chronic diseases DALYs of earlier cohorts show more rapid growth trend with age than the recent cohorts but the diseases will be developed in a later age. This indicates cohort effect is also significant for chronic diseases DALYs which is consistent with former researches that focus on elderly health. Cohort represents the life course that individuals live through (The life course of four cohorts in this study were presents in the appendix Table S1). In China, for the olds of earlier cohorts, they often suffered more negative events in their life course. For example, individuals from cohort 1942–1948 suffered wars (1937–1949) in their children stage and encountered the great famine (1959–1961) in the youth and then live 10 years through the political turmoil (the Great Cultural Revolution, 1966–1976). However, the youngest cohort in this study (cohort 1960–1965) only suffered the political turmoil in their child stage. According to the accumulation of risk model, effects accumulate over the life course, as results health damage will increase with the duration and/or number of detrimental exposures [46]. In this way, earlier cohorts have a higher DALYs than recent cohorts. Compared with the earlier, recent cohorts are generally richer and own a higher life quality, which are often more likely to have unhealthy diets, to be obese, to smoke and drink more, and to be sedentary [47, 48]. This explained reasons why the chronic diseases are developed earlier than individuals in older cohorts.

In addition, this study also finds DALYs of cohort 1954–1959 have lower growth trend than cohort 1947–1953. Individuals from these cohorts have similarly detrimental exposures which indicates they should also have the similar growth trend. This differential can be explained by the theory of critical period model which stressed the critical role of timing of one exposure [49]. Difference between these cohorts is the time they suffered the Great Cultural Revolution. For individuals in cohort 1948–1953, the Great Cultural Revolution happened in their 13 to 18 years old and lasted to their 23 to 28 years old. This period was a critical period for their life development. However, for those in cohort 1954–1959, this negative event happened in their late childhood, and last to their 17 to 22. Though influenced by this event, most of people caught up with the series reforms after the political turmoil, which reduced the negative effect to some degree [50].

Besides cohort disparity, our study finds there exists resident place, education level and income differentials for the age trajectory of chronic diseases DALYs and these disparities are enlarged/ diverged with age. This is consistent with existing researches and supported by the cumulative disadvantage/advantage theory: the positive effect of higher SES is cumulative increased by influencing recourses such as access to healthcare, health behavior and social support [51]. At the same time, it is also found these SES disparities are narrowed/converged with cohort turning younger. This can be explained by the efforts that government made to improve social medical insurance. In the last 10 years, China’s social medical insurance cover rate has been greatly improved so that individuals from younger cohort are mostly benefited [52]. On the other hand, with the urbanization, income and education level improved, SES disparities among recent cohorts are gradually narrowed [53].

Besides, it is also found resident place and education level disparities of DALYs across cohort have gender differentials. For younger female, in the last decades, their education level is improved evidently, and the government greatly improved the maternal health care especially in the rural area [54]. As results, DALYs disparities of these SES indicators are significantly narrowed across cohort. This also indicates improvements of education level and health care will have more benefit for female.

This study has several limitations. Firstly, DALYs is the sum of YLLs and YLDs, but because chronic diseases are always chronic non-fatal diseases of which the DALYs are mainly is the YLDs. In this article, we regarded the YLDs of elderly’s chronic diseases as their DALYs. Secondly, data used in this study was from 2011 to 2018, but the disability weights were from the GBD study 2013. Though the disability weights have a high universality, but it would be better to have the weight values of 2011, 2015 and 2018 which were lacked in real. Then, if mortality and non-response are significantly associated with higher DALYS, non-random selection may occur, resulting in a biased sample estimate of the trajectory. Even though we used the dummy variables of death and loss to follow-up. But it will inevitably be led to a distortion of the authenticity of DALYS calculation results. Finally, in order to examine the cohort effect from the life course perspective, the cohort in our study was limited from 1942 to 1965, which are not the full data.

Despite these limitations, this study has some advantages. Firstly, the longitudinal design of CHARLS allowed for more accumulation of information on age than single cross-sectional study. Secondly, this study is the first to measure 13 chronic diseases by calculating their DALYs, which can provide more precise and comprehensive understand of the chronic diseases’ growth trajectory in old stage.

## Conclusion

(1) DALYs of chronic diseases for middle-aged and elderly people present a general growing trend with age. Individuals having more negative event exposures in their life course show a more rapid growth trend and the timing of turmoil will also affect the DALYs trajectory. The effect of life course on chronic diseases is accumulated with exposures and its exposure in critical period is also forceful.

(2) Chronic diseases of individuals in recent cohorts are developed earlier than early cohorts. In developing countries like China, individuals in recent cohort have higher SES, but tend to have unhealthy life behaviors.

(3) Living in urban, owning higher education level and income will decrease the DALYs of chronic diseases, and advantages brought by these SES indicators will be accumulated with age. Government efforts in improving social medical insurance will lower the SES disparity which greatly benefit individuals from recent cohorts. Thus, the SES disparities show a narrowing trend with cohort turning younger.

(4) Compared with male, improvements of education level, health care and life quality will more benefit the female. The SES disparities of resident place and education level is narrowed with cohort turning younger for female. However, there no such trend for male. Governments should pay more attentions for male’s chronic diseases.

## Supplementary Information

**Additional file 1.**

## Data Availability

CHARLS data are available at http://charls.pku.edu.cn/ (requiring a simple application).

## Abbreviation

DALYs: Disabled Adjusted Life Years
