# Supplementary material for: Trajectories of middle-aged and elderly people’s chronic diseases Disability Adjusted Life Years (DALYs): cohort, socio-economic status and gender disparities
Source: Int J Equity Health. 2021 Aug 3;20:179. doi: 10.1186/s12939-021-01517-z (PMC8335861; doi:10.1186/s12939-021-01517-z)
Supplement: Supplementary file 1 — Additional file 1. [file 12939_2021_1517_MOESM1_ESM.docx]

**Table S1 Life course for cohort 1942-1947, 1948-1953, 1954-1959 and 1960-1964**

| 1942 |  | **Cohort 1942-1947** | | |  |  |  | **Cohort 1948-1953** | | |  |  |  | **Cohort 1954-1959** | | |  |  | **Cohort 1960-1964** | | |  |
| --- | --- | --- | --- | --- | --- | --- | --- | --- | --- | --- | --- | --- | --- | --- | --- | --- | --- | --- | --- | --- | --- | --- |
| 1943 | 1943 |  |  |  |  |  |  |  |  |  |  |  |  |  |  |  |  |  |  |  |  |  |
| 1944 | 1944 | 1944 |  |  |  |  |  |  |  |  |  |  |  |  |  |  |  |  |  |  |  |  |
| 1945 | 1945 | 1945 | 1945 |  |  |  |  |  |  |  |  |  |  |  |  |  |  |  |  |  |  |  |
| 1946 | 1946 | 1946 | 1946 | 1946 |  |  |  |  |  |  |  |  |  |  |  |  |  |  |  |  |  |  |
| 1947 | 1947 | 1947 | 1947 | 1947 | 1947 |  |  |  |  |  |  |  |  |  |  |  |  |  |  |  |  |  |
| 1948 | 1948 | 1948 | 1948 | 1948 | 1948 | 1948 |  |  |  |  |  |  |  |  |  |  |  |  |  |  |  |  |
| 1949 | 1949 | 1949 | 1949 | 1949 | 1949 | 1949 | 1949 |  |  |  |  |  |  |  |  |  |  |  |  |  |  |  |
| 1950 | 1950 | 1950 | 1950 | 1950 | 1950 | 1950 | 1950 | 1950 |  |  |  |  |  |  |  |  |  |  |  |  |  |  |
| 1951 | 1951 | 1951 | 1951 | 1951 | 1951 | 1951 | 1951 | 1951 | 1951 |  |  |  |  |  |  |  |  |  |  |  |  |  |
| 1952 | 1952 | 1952 | 1952 | 1952 | 1952 | 1952 | 1952 | 1952 | 1952 | 1952 |  |  |  |  |  |  |  |  |  |  |  |  |
| 1953 | 1953 | 1953 | 1953 | 1953 | 1953 | 1953 | 1953 | 1953 | 1953 | 1953 | 1953 |  |  |  |  |  |  |  |  |  |  |  |
| **1954** | 1954 | 1954 | 1954 | 1954 | 1954 | 1954 | 1954 | 1954 | 1954 | 1954 | 1954 | 1954 |  |  |  |  |  |  |  |  |  |  |
| 1955 | **1955** | 1955 | 1955 | 1955 | 1955 | 1955 | 1955 | 1955 | 1955 | 1955 | 1955 | 1955 | 1955 |  |  |  |  |  |  |  |  |  |
| 1956 | 1956 | **1956** | 1956 | 1956 | 1956 | 1956 | 1956 | 1956 | 1956 | 1956 | 1956 | 1956 | 1956 | 1956 |  |  |  |  |  |  |  |  |
| 1957 | 1957 | 1957 | **1957** | 1957 | 1957 | 1957 | 1957 | 1957 | 1957 | 1957 | 1957 | 1957 | 1957 | 1957 | 1957 |  |  |  |  |  |  |  |
| 1958 | 1958 | 1958 | 1958 | **1958** | 1958 | 1958 | 1958 | 1958 | 1958 | 1958 | 1958 | 1958 | 1958 | 1958 | 1958 | 1958 |  |  |  |  |  |  |
| 1959 | 1959 | 1959 | 1959 | 1959 | **1959** | 1959 | 1959 | 1959 | 1959 | 1959 | 1959 | 1959 | 1959 | 1959 | 1959 | 1959 | 1959 |  |  |  |  |  |
| **1960** | 1960 | 1960 | 1960 | 1960 | 1960 | **1960** | 1960 | 1960 | 1960 | 1960 | 1960 | 1960 | 1960 | 1960 | 1960 | 1960 | 1960 | 1960 |  |  |  |  |
| 1961 | **1961** | 1961 | 1961 | 1961 | 1961 | 1961 | **1961** | 1961 | 1961 | 1961 | 1961 | 1961 | 1961 | 1961 | 1961 | 1961 | 1961 | 1961 | 1961 |  |  |  |
| 1962 | 1962 | **1962** | 1962 | 1962 | 1962 | 1962 | 1962 | **1962** | 1962 | 1962 | 1962 | 1962 | 1962 | 1962 | 1962 | 1962 | 1962 | 1962 | 1962 | 1962 |  |  |
| 1963 | 1963 | 1963 | **1963** | 1963 | 1963 | 1963 | 1963 | 1963 | **1963** | 1963 | 1963 | 1963 | 1963 | 1963 | 1963 | 1963 | 1963 | 1963 | 1963 | 1963 | 1963 |  |
| 1964 | 1964 | 1964 | 1964 | **1964** | 1964 | 1964 | 1964 | 1964 | 1964 | **1964** | 1964 | 1964 | 1964 | 1964 | 1964 | 1964 | 1964 | 1964 | 1964 | 1964 | 1964 | 1964 |
| 1965 | 1965 | 1965 | 1965 | 1965 | **1965** | 1965 | 1965 | 1965 | 1965 | 1965 | **1965** | 1965 | 1965 | 1965 | 1965 | 1965 | 1965 | 1965 | 1965 | 1965 | 1965 | 1965 |
| 1966 | 1966 | 1966 | 1966 | 1966 | 1966 | **1966** | 1966 | 1966 | 1966 | 1966 | 1966 | **1966** | 1966 | 1966 | 1966 | 1966 | 1966 | 1966 | 1966 | 1966 | 1966 | 1966 |
| **1967** | 1967 | 1967 | 1967 | 1967 | 1967 | 1967 | **1967** | 1967 | 1967 | 1967 | 1967 | 1967 | **1967** | 1967 | 1967 | 1967 | 1967 | 1967 | 1967 | 1967 | 1967 | 1967 |
| 1968 | **1968** | 1968 | 1968 | 1968 | 1968 | 1968 | 1968 | **1968** | 1968 | 1968 | 1968 | 1968 | 1968 | **1968** | 1968 | 1968 | 1968 | 1968 | 1968 | 1968 | 1968 | 1968 |
| 1969 | 1969 | **1969** | 1969 | 1969 | 1969 | 1969 | 1969 | 1969 | **1969** | 1969 | 1969 | 1969 | 1969 | 1969 | **1969** | 1969 | 1969 | 1969 | 1969 | 1969 | 1969 | 1969 |
| 1970 | 1970 | 1970 | **1970** | 1970 | 1970 | 1970 | 1970 | 1970 | 1970 | **1970** | 1970 | 1970 | 1970 | 1970 | 1970 | **1970** | 1970 | 1970 | 1970 | 1970 | 1970 | 1970 |
| 1971 | 1971 | 1971 | 1971 | **1971** | 1971 | 1971 | 1971 | 1971 | 1971 | 1971 | **1971** | 1971 | 1971 | 1971 | 1971 | 1971 | **1971** | 1971 | 1971 | 1971 | 1971 | 1971 |
| 1972 | 1972 | 1972 | 1972 | 1972 | **1972** | 1972 | 1972 | 1972 | 1972 | 1972 | 1972 | **1972** | 1972 | 1972 | 1972 | 1972 | 1972 | **1972** | 1972 | 1972 | 1972 | 1972 |
| 1973 | 1973 | 1973 | 1973 | 1973 | 1973 | **1973** | 1973 | 1973 | 1973 | 1973 | 1973 | 1973 | **1973** | 1973 | 1973 | 1973 | 1973 | 1973 | **1973** | 1973 | 1973 | 1973 |
| 1974 | 1974 | 1974 | 1974 | 1974 | 1974 | 1974 | **1974** | 1974 | 1974 | 1974 | 1974 | 1974 | 1974 | **1974** | 1974 | 1974 | 1974 | 1974 | 1974 | **1974** | 1974 | 1974 |
| 1975 | 1975 | 1975 | 1975 | 1975 | 1975 | 1975 | 1975 | **1975** | 1975 | 1975 | 1975 | 1975 | 1975 | 1975 | **1975** | 1975 | 1975 | 1975 | 1975 | 1975 | **1975** | 1975 |
| 1976 | 1976 | 1976 | 1976 | 1976 | 1976 | 1976 | 1976 | 1976 | **1976** | 1976 | 1976 | 1976 | 1976 | 1976 | 1976 | **1976** | 1976 | 1976 | 1976 | 1976 | 1976 | **1976** |
| 1977 | 1977 | 1977 | 1977 | 1977 | 1977 | 1977 | 1977 | 1977 | 1977 | **1977** | 1977 | 1977 | 1977 | 1977 | 1977 | 1977 | **1977** | 1977 | 1977 | 1977 | 1977 | 1977 |
| 1978 | 1978 | 1978 | 1978 | 1978 | 1978 | 1978 | 1978 | 1978 | 1978 | 1978 | **1978** | 1978 | 1978 | 1978 | 1978 | 1978 | 1978 | **1978** | 1978 | 1978 | 1978 | 1978 |
| 1979 | 1979 | 1979 | 1979 | 1979 | 1979 | 1979 | 1979 | 1979 | 1979 | 1979 | 1979 | **1979** | 1979 | 1979 | 1979 | 1979 | 1979 | 1979 | **1979** | 1979 | 1979 | 1979 |
| 1980 | 1980 | 1980 | 1980 | 1980 | 1980 | 1980 | 1980 | 1980 | 1980 | 1980 | 1980 | 1980 | **1980** | 1980 | 1980 | 1980 | 1980 | 1980 | 1980 | **1980** | 1980 | 1980 |
| 1981 | 1981 | 1981 | 1981 | 1981 | 1981 | 1981 | 1981 | 1981 | 1981 | 1981 | 1981 | 1981 | 1981 | **1981** | 1981 | 1981 | 1981 | 1981 | 1981 | 1981 | **1981** | 1981 |
| 1982 | 1982 | 1982 | 1982 | 1982 | 1982 | 1982 | 1982 | 1982 | 1982 | 1982 | 1982 | 1982 | 1982 | 1982 | **1982** | 1982 | 1982 | 1982 | 1982 | 1982 | 1982 | **1982** |

Note: (1) Orange area represent the War time; Blue area represent the great famine; Green area represent the Cultural Revaluation.

(2) There are three red line which represent 12-year-old, 18-year-old and 25-year-old.

**Table S2 Comparison of growth models fitting values**

|  | AIC | BIC | -2LL |
| --- | --- | --- | --- |
| Line growth model | 89381.2 | 89402.1 | 89373.4 |
| Quadratic curve model | 89369.5 | 89397.7 | 89361.5 |
| Cubic growth model | 89403.4 | 89428.5 | 89407.3 |
| The cubic spline model | 89603.9 | 89630.5 | 89602.4 |

Note: Hierarchical linear growth model of predicted trajectories of DALYs score from 45 to 90 years of all participates, predicted from a random coefficient and random slope model. AIC: Akaike Information Criterion. BIC: Bayesian Information Criterion. LL: the log likelihood.
